# Premenstrual dysphoric disorder and childhood maltreatment, adulthood stressful life events and depression among Lebanese university students: a structural equation modeling approach

**DOI:** 10.1186/s12888-021-03567-7

**Published:** 2021-11-09

**Authors:** Yorgo Younes, Souheil Hallit, Sahar Obeid

**Affiliations:** 1 Faculty of Arts and Sciences, Holy Spirit University of Kaslik (USEK), Jounieh, Lebanon; 2 Research Department, Psychiatric Hospital of the Cross, Jal Eddib, Lebanon; 3 Faculty of Medicine and Medical Sciences, Holy Spirit University of Kaslik (USEK), Jounieh, Lebanon

**Keywords:** Premenstrual dysphoric disorder, Depression, Childhood maltreatment, life’s stressful events, Lebanese women

## Abstract

**Background:**

Premenstrual Syndrome (PMS) is a cyclic sequence of physical and behavioral symptoms that arise in the second half of the menstrual cycle. The extreme type of PMS is Premenstrual Dysphoric Disorder (PMDD). The current study aims at examining 1) the effects of childhood maltreatment and current life’s stressful events on PMDD, and 2) the mediating role of depression in these associations among Lebanese university female students.

**Methods:**

This cross-sectional study was conducted between February and March 2021 during the COVID-19 pandemic. Lebanese students were recruited using a snowball technique from all national universities in Lebanon via an auto-administrated online survey. Structural equation modeling was performed to examine the structural relationship between childhood maltreatment and life’s stressful events, depression and PMDD.

**Results:**

Higher life’s stressful events (Beta = 0.18; *p* < 0.001), physical (Beta = 0.19; *p* < 0.001), sexual (Beta = 0.18; *p* < 0.001) and psychological (Beta = 0.33; *p* < 0.001) abuse were significantly associated with higher depression. Moreover, higher sexual (Beta = 0.11; *p* = 0.021) and psychological (Beta = 0.11; *p* = 0.040) abuse and higher depression (Beta = 0.37; *p* < 0.001) were significantly associated with higher PMDD. The indirect relationships between psychological abuse/sexual abuse, depression and PMDD showed that depression mediated the association between both psychological (Beta = 0.22; *p* = 0.001) and sexual (Beta = 0.38; *p* = 0.004) abuse and PMDD.

**Conclusion:**

This work presents a unique analysis using the structural equation model that enlightens the effect of childhood maltreatment, particularly sexual and psychological abuse on PMMD symptoms, with depression playing the role of a mediating factor. It would be interesting to test, in future studies, whether there are other mediating factors besides depression that could be indirect indicators of PMDD.

## Introduction

Premenstrual Syndrome (PMS) is a cyclic sequence of physical and behavioral symptoms that arise in the second half of the menstrual cycle. The extreme type of PMS is Premenstrual Dysphoric Disorder (PMDD). The 5th edition of the Diagnostic and Statistical Manual of Mental Disorders (DSM-5) [1] defines Premenstrual Dysphoric Disorder (PMDD) as a depressive disorder ranging from 1.8 to 5.8% among women who menstruate, within a 12-month prevalence. PMDD is characterized by affective and physical signs that are close to those of Major Depressive Disorder (MDD) and manifest during the last week of the luteal phase of the menstrual period and vanish immediately after menstruation starts [2].

According to the DSM-5 [1], PMDD is usually diagnosed when 5 out of the following 11 symptoms are present during the last week of the luteal phase. Those symptoms should not represent amplification of preexisting depression, personality disorder or anxiety such as depressed mood, anxiety, affective lability (feeling suddenly sad or tearful or increased sensitivity to rejection), persistent irritability or increase in interpersonal conflicts, decreased interest in usual activities, lack of energy, change in appetite, sleep difficulties, personal sense of being overwhelmed or other physical symptoms like headaches, breast tenderness or swelling, weight gain, etc.

Menstruation-related symptoms can adversely impact a woman’s life and have a direct impact on productivity [2]. PMDD triggers a spike in absenteeism at work [3], as well as a decrease in productivity and quality of life [3, 4]. Moreover, a recent study [5] found that women with PMDD perceived everyday stressors as more aversive, with a substantial rise in high-arousal negative affect states in the late luteal phase of the menstrual cycle, relative to the follicular phase when compared to healthy controls.

Studies show that several factors might be associated with PMDD, including psychosocial factors, such as child psychological, physical, and sexual maltreatment [6]. The Word Health Organization (WHO) [7] defines childhood maltreatment as abuse and neglect of minors under the age of 18. It encompasses all forms of physical and/or emotional violence, as well as sexual abuse, neglect, negligence, and commercial or other forms of exploitation that cause real or potential harm to a child’s health, life, growth, or dignity in the context of a relationship of trust, control or responsibility. Several studies have found that people with PMDD have a history of childhood maltreatment. For instance, women with PMDD were 6.7 times more likely to report childhood sexual assault than controls [8]. Moreover, childhood maltreatment raises the risk of PMDD later in life [6]. In addition, when compared to healthy controls, women living with PMDD were found more likely to have undergone childhood trauma, such as mental distress and/or abandonment, physical and/or sexual abuse [9, 10]. Also, women that have suffered from abuse in the past are more likely to show serious premenstrual symptomatology [6, 11]. It was also documented that childhood maltreatment, particularly neglect, can represent an indirect predictor of PMDD symptoms [12].

Adding to childhood maltreatment, adult life’s stressful events play a role in PMDD. Stressful life events are described as experiences that were likely to cause readjustment in people’s regular activities [13], such as death of a spouse, divorce, major personal injury of illness, pregnancy, etc. [14]. Previous studies [2, 15] highlighted that adult stressful life’s experiences are a main example of environmental and psychological factors that can lead to depressive symptoms or major depressive disorders, including PMDD.

A variety of experiments has looked at the temporal association between PMDD and major depression. It is of note that PMDD and depression share common symptoms, which leads to a difficult distinction between them [16]. Some scholars stated that irritability and mood swings are included in measures for both disorders [17], while others emphasized that, considering their striking similarity, PMDD and depression should be seen as separate psychiatric entities [18]. Irritability has been identified as a more common symptom in women with PMDD rather than depression [19]. Moreover, previous findings concluded that the distinction between the two disorders can be observed through differences in the dysregulation in the stress axes in women [20]. Moreover, the results of research on risk factors for depression and PMDD revealed that the two conditions tend to have different causes; premenstrual symptoms appear to be affected by familial-environmental factors either to a limited degree or not at all, while depression was affected to a more pertinent degree [21]. Regarding the correlation between these two variables, results are controversial. Previous authors [22, 23] found that women with PMDD have a greater rate of previous severe depression than women without PMDD. However, Forrester-Knauss et al. [24] found that major depression was only observed in 11.3% of women with mild PMS and 24.6% of those with PMDD.

Adding to this direct correlation between PMDD and depression, and as mentioned previously, stressful life’s events have been consistently associated with an increase in depressive symptoms [25] and the onset of major depression in adults [26]. Moreover, previous results [27] suggested a substantial influence of multiple childhood trauma on a severe and chronic course of depression in adulthood. Patients reporting multiple childhood trauma showed greater symptoms’ severity, suggesting a dose-response relationship between the number of childhood maltreatments and symptomatology. In addition, the number and severity of premenstrual symptoms increase with more exposure to childhood trauma, with this relationship being completely mediated by emotion regulation difficulties [28] (depression being a disorder of impaired emotion regulation [29]). These different correlations could suggest that childhood maltreatment and stressful life events have an effect on the prevalence of PMDD, with depression playing the role of a mediating factor in these associations.

Among Lebanese women, Costanian et al. (2018) [30] noted that PMS was reported by 63% of participants, of which 42.5% having severe PMS (or PMDD). However, high depressive symptoms were prevalent among 59.7% of Lebanese [31], while 30% of Lebanese children reported at least one experience of witnessing violence, 65% reported at least one incident of psychological abuse, 54% reported at least one incident of physical abuse [32] and 16.1% reported going through at least one experience of sexual abuse [33]. During the past couple of years, women living in Lebanon experienced many stressful events ranging from economic instability, to lockdowns caused by the COVID-19 pandemic, in addition to the Beirut Port explosion [34]. The country is also going through a severe economic crisis in which unemployment rate has reached around 30% mark estimated by a Lebanese consulting firm [35]. Moreover, social stressful life’s events, in addition to health issues and witnessed stressful events, were found to predict PTSD and depression among Lebanese [36]. It is important to note herein that there have been no studies in Lebanon, which have looked at the connection between PMDD, childhood maltreatment, adult life’s stressful events and depression. As a result, the current study aims at examining 1) the effects of childhood maltreatment and current stressful events on PMDD, and 2) the mediating role of depression in the associations among Lebanese university female students.

## Methods

### Study design and procedure

This cross-sectional study was conducted between February and March 2021 during the COVID-19 pandemic and the lockdown period imposed by the Lebanese government. Lebanese students were recruited using the snowball technique from all national Universities in Lebanon via an auto-administrated online survey. Participants were above 18 years; excluded were those who refused to participate in the study.

### Minimal sample size calculation

Based on a mean physical abuse score of 23.80 ± 5.51 in healthy controls and 26.48 ± 6.79 in those with PMDD [10], a power of 95% and a risk of error of 5%, the G-power calculated a minimal sample of 232 female university students to be enrolled.

### Questionnaire and variables

The Arabic self-administered questionnaire, which required approximately 30 min to fill, was anonymous and contained close-ended questions. It was divided to several sections. The first part tackled sociodemographic characteristics including age, educational level, marital status, and household crowding index. The latter was calculated by dividing the number of people in the house by the number of rooms, except the bathrooms and kitchen [37]. The physical activity index was determined by multiplying together the intensity, the time and the frequency of physical activity [38]. Moreover, some questions were dedicated to observe the menstrual activity of the respondents including the number of days between each menstrual cycle, in addition to whether the cycle is usually regular or not. The second part of the questionnaire included the following scales:

#### Premenstrual symptoms screening tool

The Premenstrual Symptoms Screening Tool (PSST), which is a retrospective questionnaire, helps distinguish women who experience severe PMDD [16]. Women use a 4-point Likert scale to measure the magnitude of their symptoms over the previous year (1 = none, 2 = mild, 3 = moderate, 4 = severe). The PMDD scale is divided into two parts, the first of which contains 12 PMDD symptoms. If the respondent selects “yes” for at least one symptom (i.e. 2–4 points), she must complete section B, which consists of five questions about menstruation-related disturbance of activities, behaviors or relationships. The total score is calculated by summing the answers to the 17 items (range between 17 and 68). In this study, the Cronbach’s alpha was 0.936.

#### The Holmes-Rahe life stress inventory

The Holmes-Rahe Life Stress inventory is a self-administered scale used for measuring the amount of stress someone has experienced within the past year [14]. It consists of 43 items tackling life experiences such as death of close family member, being fired at work, pregnancy, sexual difficulties, etc. A score of 150 points or less indicates a low susceptibility to stress-induced health breakdowns. A score between 150 and 300 points implies a 50% chance of a major health breakdown in the coming 2 years, while scoring more than 300 points raises the odds to 80%. In this study, the Cronbach’s alpha was 0.748.

#### The Lebanese depression inventory (LDS-19)

The LDS-19 scale evaluates depression in adults during the last 2 weeks (including the interview day) and requires 10 min to complete. The total score is the sum of all answers, with higher scores indicating higher depression [39]. In this study, the Cronbach’s alpha was 0.801.

#### Child abuse self-report scale (CASRS)

This scale tackles 38 items divided into four categories: psychological (14 items), neglect (11 items), physical (8 items) and sexual (5 items) [40]. The responses are scored from 0 = Never to 3 = Always. In all subscales, higher scores point out more abuse [41]. In this study, the Cronbach’s alpha values were as follows: psychological (0.933), neglect (0.902), physical (0.878), and sexual (0.893). The Arabic version of this scale was used in previous papers [42–46].

### Translation procedures

All scales, except the LDS and the CASRS, were translated from English to Arabic using one bilingual translator. A backward translation was then performed by a native English-speaking translator, fluent in Arabic and unfamiliar with the concepts of the scales. Minor discrepancies were solved by consensus.

### Statistical analysis

All analyses were carried out using the Statistical Package for Social Sciences (version 24.0 with AMOS; IBM®, Armonk, NY, U.S.A.). We had no missing values in our database since all questions were required in the Google form. Structural equation modeling (SEM) was performed to examine the structural relationship between childhood maltreatment and life’s stressful events taken as independent variables, PMDD as the dependent variable, and depression as the mediator, among university students. In the first step of the SEM, the assessment of normality of the PMDD score was performed by calculation of the skewness and kurtosis; values for asymmetry and kurtosis between − 1 and + 1 are considered acceptable in order to prove normal univariate distribution [47]. These conditions consolidate the assumptions of normality in samples larger than 300 [48]. The root mean square error of approximation (RMSEA) statistic, Tucker Lewis Index (TFI) and the comparative fit index (CFI) were used to evaluate the goodness-of-fit of the model [31]. RMSEA values ≤0.06 or CFI and TFI values > 0.90 indicate a good-fitting model [31]. *P* < 0.05 was considered statically significant.

## Results

The mean age of the participants was 21.75 ± 4.78 years, with a mean PMDD score of 30.83 ± 13.17. Other description of the sample can be found in Table 1.

**Table 1:** Sociodemographic and other characteristics of the participants (*N* = 318)

Variable	N (%)
District	
Beirut	53 (16.7%)
Mount Lebanon	191 (60.1%)
North	24 (7.5%)
South	23 (7.2%)
Bekaa	27 (8.5%)
Marital status	
Single	302 (95.0%)
Married	16 (5.0%)
Regular menses	
No	220 (69.2%)
Yes	98 (30.8%)
Oral contraceptives	
No	297 (93.4%)
Yes	21 (6.6%)
**Mean ± SD**	
Age (in years)	21.75 ± 4.78 (Min = 18; Max = 30)
Household crowding index	0.95 ± 0.42 (Min = 0; Max = 4)
Physical activity index	25.50 ± 18.77 (Min = 1; Max = 100)
Life’s stressful events score	249.67 ± 133.53 (Min = 0; Max = 702)
Depression score	17.50 ± 8.21 (Min = 3; Max = 41)
Psychological abuse score	5.28 ± 7.20 (Min = 0; Max = 42)
Neglect score	24.19 ± 7.16 (Min = 0; Max = 33)
Physical abuse score	1.00 ± 2.38 (Min = 0; Max = 18)
Sexual abuse score	1.13 ± 2.27 (Min = 0; Max = 15)
Premenstrual dysphoric disorder (PMDD) score	30.83 ± 13.17 (Min = 0; Max = 56)

### Structural equation modeling

**Fig. 1:**
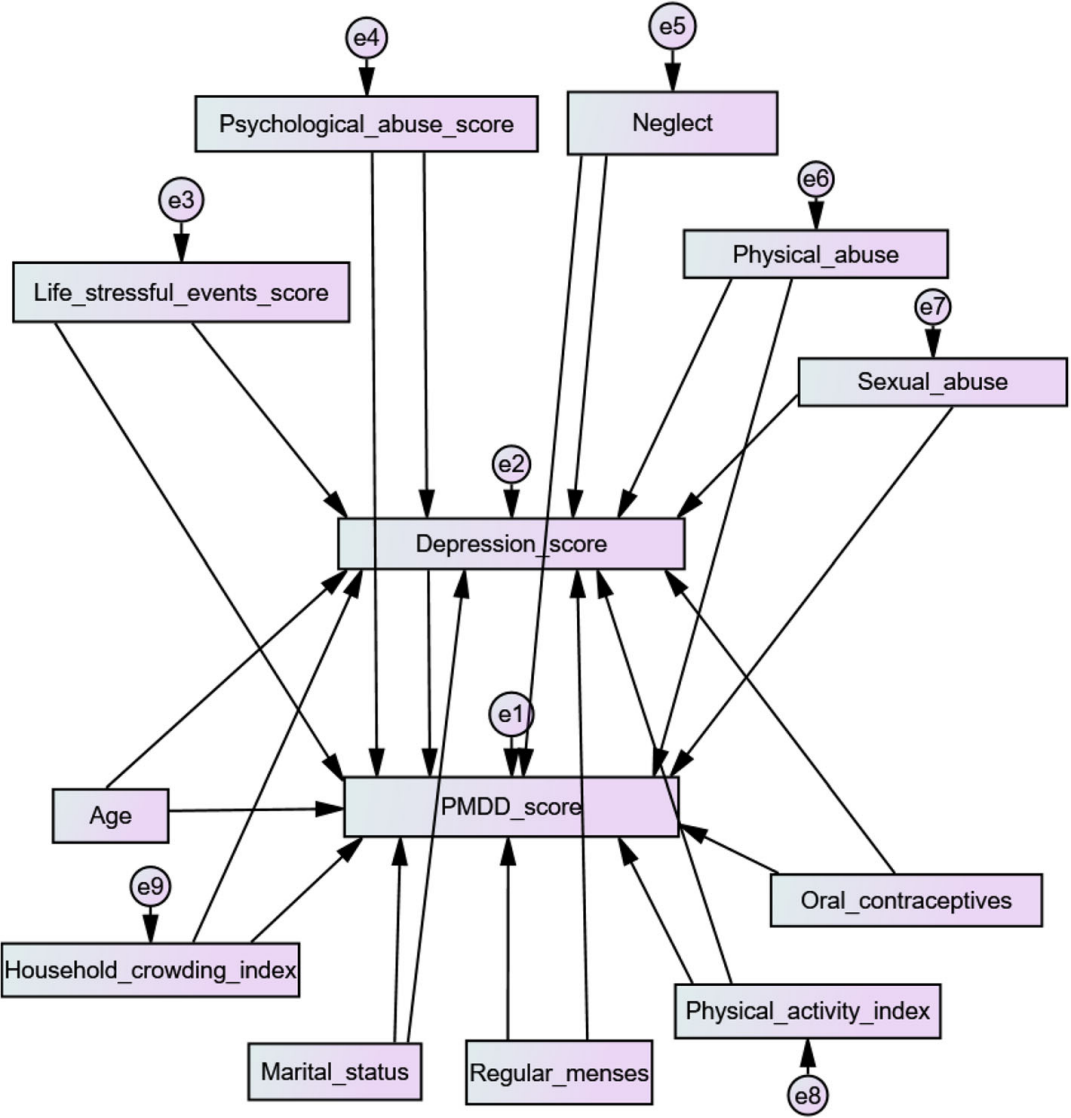
Structural equation model in university students. —observed variable; —latent variable; →—impact of one variable on another; e—residual error in the prediction of an unobserved factor

The structural relationships between childhood maltreatment and life’s stressful events taken as independent variables, PMDD as the dependent variable, and depression as the mediator, are shown in Fig. 1. There was no multicollinearity between the variables entered in the model. The RMSEA, TFI and CFI values were 0.023 (pclose = 0.722), 0.92 and 0.98 respectively, indicating a good fit of the model. Higher life’s stressful events (Beta = 0.18; *p* < 0.001), physical (Beta = 0.19; *p* < 0.001), sexual (Beta = 0.18; *p* < 0.001) and psychological (Beta = 0.33; *p* < 0.001) abuse were significantly associated with higher depression. Moreover, higher sexual (Beta = 0.11; *p* = 0.021) and psychological (Beta = 0.11; *p* = 0.040) abuse and higher depression (Beta = 0.37; *p* < 0.001) were significantly associated with higher PMDD. The indirect relationships between psychological abuse/sexual abuse, depression and PMDD showed that depression mediated the association between both psychological (Beta = 0.22; *p* = 0.001) and sexual (Beta = 0.38; *p* = 0.004) abuse and PMDD.

Table 2 presents the standardized coefficients with standard errors and *p*-values of the direct effects of variables on each other.

**Table 2:** Standardized coefficient, standard error and *p*-value of the structured equation modeling (SEM)

Variable	Standardized Coefficient	Standard error	***P***
**PMDD (dependent variable)**			
Sexual abuse	0.11	0.29	**0.021**
Life stressful events	0.06	0.01	0.209
Psychological abuse	0.11	0.09	**0.040**
Physical abuse	−0.01	0.27	0.835
Household crowding index	0.07	1.49	0.130
Marital status (married vs single*)	0.02	2.88	0.713
Regular menses (yes vs no*)	0.05	1.40	0.299
Physical activity index	−0.08	0.04	0.113
Oral contraceptives intake (yes vs no*)	−0.01	2.57	0.801
Age	−0.03	0.13	0.568
Neglect	0.002	0.09	0.960
Depression	0.37	0.09	**< 0.001**
**Depression (dependent variable)**			
Life stressful events	0.18	0.003	**< 0.001**
Physical abuse	0.19	0.16	**< 0.001**
Sexual abuse	0.18	0.17	**< 0.001**
Neglect	−0.09	0.05	0.061
Psychological abuse	0.33	0.05	**< 0.001**
Age	−0.03	0.08	0.502
Household crowding index	−0.02	0.89	0.635
Marital status (married vs single*)	0.001	1.72	0.997
Regular menses (yes vs no*)	0.20	0.82	**< 0.001**
Oral contraceptives intake (yes vs no*)	−0.13	1.52	**0.006**
Physical activity index	−0.25	0.02	**< 0.001**

Numbers in bold indicate significant *p*-values

## Discussion

To our knowledge, this is the first study in Lebanon showing that higher sexual and psychological abuse are directly associated with higher PMDD, with these associations being mediated by depression according to the structural equation modeling.

### Childhood maltreatment and depression

In terms of association between physical abuse, neglect and depression, our research revealed that more physical abuse and neglect were associated with higher depression, which supports several previous findings. According to a recent study [49], a history of childhood maltreatment was associated with depression in adolescents. All dimensions of childhood maltreatment had a significant association with depression, with psychological violence having the most impact on the occurrence of depression, followed by exposure to violence and physical violence. Moreover, Spinhoven et al. [50] found that emotional neglect is a predicting factor of depression.

### Stressful life’s events and depression

Results of our study showed that stressful life’s events were associated with higher depression but not PMDD. This finding supports previous research [2, 15, 31] that highlighted that adulthood stressful life’s events are one of the most common environmental and psychological causes that may trigger depressive symptoms or major depressive disorders. Furthermore, people with a history of notable emotional trauma reported greater rises in depressive symptoms when faced with contingent stressful life’s events [25]. The occurrence of negative life’s events, as well as one’s perception of/response to those events, can influence the production and persistence of depressive symptoms, according to cognitive-behavioral models of depression [51]. Individuals that have a propensity to create negative attributions in relation to the triggers of negative life experiences, about themselves, and about potential outcomes (in accordance with the hopelessness theory of depression) might be more prone to experiencing depression, according to cognitive vulnerability-stress models [52].

In relation to the Lebanese context, recent findings showed that 28.9% of Lebanese adolescents had suicidal ideation [53], 31.7% were alexithymic and 26% had depression in its severe forms [54]. Moreover, 28.9% of Lebanese adults had suicidal ideation [44], 20.8% had alexithymia [55], 32.9 and 26.9% suffered from mild-to-moderate and severe -to-very severe depression [31], whereas 19.9 and 18.4% had moderate and severe anxiety [56] respectively. Such numbers reveal the severity of the situation in Lebanon when it comes to mental health. Our study, which showed the direct effect of major stressful life’s events (such as the pandemic, Beirut Port explosion, and change in the economic situation) on depression, can provide insight to health care providers when dealing with patients directly affected by those major events.

### Childhood maltreatment, PMDD and depression

In our study, sexual and psychological abuse were positively associated with the severity of PMDD symptoms among Lebanese university students. These findings support previous research that suggest that childhood maltreatment may play a role in the development of PMDD [57]. Furthermore, patients with PMDD had a higher overall score on the Childhood Trauma Questionnaire, as well as higher scores for physical, emotional, and sexual abuse than healthy controls, according to previous authors [10]. Bertone-Johnson et al. [6] also concluded that childhood maltreatment, with emphasis on emotional and physical abuse, raises the risk of moderate-to-severe PMS (PMDD being the severe type of PMS).

The association between sexual and psychological abuse and PMDD was mediated by depression (depression being a disorder of impaired emotion regulation [29], which is linked to the development of psychopathology later in life) [58, 59]. Emotion regulation is described as the knowledge and comprehension of one’s emotions, acceptance of emotional reactions, and how one reacts and behaves in response to these emotions [60]. Several studies have shown that emotion regulation abilities mediate the connection between childhood trauma and psychopathology. Van der Kolk & Bessel [61] found that one of the most common characteristics of children who have been subject to abuse is their failure to control their emotions in an adaptive fashion. The lack of self-regulatory mechanisms in these children contributes to weak modulated affect, decreased impulse regulation, mistrust, and confusion about others’ reliability and predictability [61, 62]. Hence, the effect of maltreatment on children’s anxious and depressive symptoms was induced by a maladaptive pattern of emotion control [63]. Furthermore, Kim & Cicchetti [64] showed that emotional and/or sexual violence in parent-child relationships are linked to an evolving aberration in the organization of affective systems, which eventually leads to psychopathology. As a result, cyclic fluctuations in mood, affect, and cognition, which happen on a monthly basis, can be more difficult for women with a history of childhood trauma, mainly due to deficiencies in emotion regulation [28]. On another note, childhood maltreatment leads to hypothalamic-pituitary-adrenal axis hyperactivity, developmental changes in the brain, and epigenetic changes in the amygdala and hippocampus. Stress reactions can be heightened as a result of these biological effects [65].

### Clinical implications

In relation to our findings, healthcare professionals need to take into consideration two main issues while assessing women with PMDD: 1) the mental state of those women, especially those who also manifest symptoms of depression (since it was shown to be a mediating factor of PMDD); and 2) the possibility of childhood maltreatment history (especially emotional and sexual abuse). By taking into account those two factors, health care professionals can assess patients more accurately, in addition to providing adequate interventions that fit their needs and their history.

Moreover, the positive correlation between stressful life’s events and depression is a great insight for healthcare professionals dealing with patients who recently experienced such events, especially in the Lebanese context. During the past couple of years, women living in Lebanon experienced many stressful events as previously mentioned. The parallel occurrence of such stressful events with the sharp rise in mental illness, confirms our findings where life’s stressful events were positively correlated with depression. Thus, healthcare professionals should take into consideration those major events in relation with depression among Lebanese women when designing their approach.

### Limitations

Several limitations can be attributed to this study. First, a screening questionnaire was used to diagnose PMDD, despite the fact that the DSM-5 states that this diagnosis involves the completion of a prospective daily rating for at least two menstrual cycles. Moreover, the PSST is a retrospective questionnaire capturing symptoms in the past year, which is considered far less accurate compared with prospective rating of mood symptoms. Additional diagnosis of PMDD, according to the DSM-5, also includes an increase of at least 30% (relative to the range of the scale used) in the mean self-ratings of negative moods (i.e., irritability, depression, anxiety, and mood swings) in the 7 days before menses as compared with the ratings for the 7 days afterward in at least two of the three base-line cycles. Nevertheless, PSST has been used in previous research since its publication [28, 66]. In addition, the International Society of Premenstrual Disorders has accepted it as a possible diagnostic tool [67]. Second, the research is focused on self-report questionnaires outlining adverse experiences that occurred in the past. Hence, information bias may be present since the data is based on self-reported answers. Due to the cross-sectional design of the study, assumptions about causality remain hypothetical. Moreover, the used scales (except the LDS) are not validated in Lebanon to date. Selection bias is possible due to the snowball technique used to enroll participants. Adding to that, residual confounding bias is also possible since not all factors associated with PMDD have been taking into consideration in this study. For example, several other symptoms (e.g. irritability, sadness, anxiety) are considered to be equally as critical in women with PMDD as depressive symptoms while only depression was used as a mediating factor in our study. Further studies should focus on using those other symptoms as mediators. Finally, the study’s sample size limits the findings’ generalizability. Future research using a longitudinal design and a larger sample could help us better understand these relationships.

## Conclusion

This work presents a unique analysis using the structural equation model that enlightens the effect of childhood maltreatment, particularly sexual and psychological abuse on the prevalence of PMMD symptoms with depression playing the role of a mediating factor. More importantly, it represents a timely investigation tackling the consequences of major stressful life’s events, physical abuse and neglect on the occurrence of depressive symptoms following rapid drastic events (including COVID-19, the explosion of the Beirut port and the Lebanese economic crisis), providing an environmental context that might be unique worldwide. It would be interesting to test, in future studies, whether there were other mediating factors besides depression that could be an indirect indicator of PMDD.

## Data Availability

Data cannot be shared publicly due to restrictions imposed by the Psychiatric Hospital of the Cross Ethics Committee to protect patient confidentiality, but is available upon request to the corresponding author.

## Abbreviations

PMS: Premenstrual Syndrome
PMDD: Premenstrual Dysphoric Disorder
DSM-5: 5th edition of the Diagnostic and Statistical Manual of Mental Disorders
MDD: Major Depressive Disorder
WHO: Word Health Organization
PSST: Premenstrual Symptoms Screening Tool
LDS: Lebanese Depression Inventory
SEM: Structural equation modeling
RMSEA: Root mean square error of approximation
TLI: Tucker Lewis Index
CFI: Comparative fit index
